# Research on intelligent design and environmental adaptability of public art installations in the context of low-carbon cities

**DOI:** 10.3389/fpubh.2025.1619585

**Published:** 2025-09-04

**Authors:** Wei Wu, He Ren

**Affiliations:** School of Art and Design, Beijing Forestry University, Beijing, China

**Keywords:** low-carbon cities, intelligent design, environmental adaptability, public art installations, multidimensional assessment

## Abstract

The idea of a low-carbon city is to achieve sustainable development by reducing greenhouse gas emissions. This study explores how intelligent design can enhance the environmental adaptability of public art installations and contribute to urban low-carbon goals. In the study, five representative public art installations were investigated by means of literature review, case analysis and empirical research. The results show that intelligent design functions (such as sensor response time ≤ 0.5 s and AI algorithm accuracy rate of 95%) significantly improve the environmental performance. The unit energy consumption is reduced to 0.35 kWh/h, renewable energy accounts for 85% of the total consumption, and the comprehensive carbon emission intensity is reduced to 0.24 kg CO_2_e/h. In addition, user satisfaction plays a key regulatory role: every time the satisfaction score increases by 1 point, the comprehensive performance score increases by 12.3 points on average. The research results show that intelligent technologies, including edge computing and artificial intelligence, can optimize energy efficiency and ecological performance. In practical application, the trade-off between technical cost, maintenance complexity and material durability must be considered. This study constructs a multi-dimensional evaluation framework based on AHP, which provides practical reference for the design and evaluation of intelligent public art facilities in a low-carbon city environment.

## 1 Introduction

Under the background of global climate change, low-carbon cities have gradually become the focus of attention of governments and academic circles in various countries. As a key strategy to deal with global warming, resource depletion and other environmental problems, the construction of low-carbon cities needs to be explored from the aspects of urban culture and public space, in addition to energy structure optimization, transportation system improvement and building energy conservation (1, 2). In this process, public art installation, as an urban element with both aesthetic value and social function, has been given a new mission (3). It is not only a symbol of urban culture, but also an important link between man and nature, technology and environment (4). Public art installations refer to large-scale and specific works of art placed in public spaces, which are usually designed to blend in with the environment and community. These devices range from interactive sculptures and digital lighting displays to dynamic structures that respond to environmental stimuli. The core of the concept of low-carbon city is to achieve sustainable development by reducing greenhouse gas emissions (5). With the acceleration of industrialization and the rising urbanization rate, the impact of human activities on the consumption of natural resources and the environment is becoming more and more serious (6). According to the statistics of the International Energy Agency (IEA), urban carbon emissions account for more than 70% of the world, and this proportion is still rising. Based on this factor, promoting the construction of low-carbon cities has become a global consensus. The goal of low-carbon cities is not only to reduce the carbon footprint, but also to improve the quality of life of residents, promote the restoration of ecosystems and build a more inclusive and resilient social structure (7).

The construction of low-carbon cities is not easy. In addition to technological innovation, it is necessary to comprehensively consider many dimensions such as culture, education and policy (8). Especially in public space design, the traditional way often ignores the importance of environmental friendliness and user participation (9). For example, although sculptures, landscape sketches and other public art installations in many cities have ornamental value, they are single in function and lack of interaction, and may even waste additional resources due to improper material selection or poor maintenance (10, 11). This leads to the failure of public art installations to give full play to their potential in the construction of low-carbon cities. Since its birth, public art installations have played a role in beautifying the city, transmitting ideas and stimulating emotions (12). From ancient Greek temple sculptures to digital interactive devices in modern cities, these works record the development of human civilization in different forms (13). With the progress of science and technology and the popularization of environmental awareness, the functions of public art installations have changed significantly. In the past, they were mostly regarded as static artistic expressions; nowadays, it is gradually integrating intelligent technology and ecological design concept, and has the ability to dynamically respond to the environment (14).

In the context of low-carbon cities, public art installations are no longer just to decorate the existence of cities, but an important tool to promote green transformation (15). On the one hand, with the help of intelligent technology, energy can be used efficiently. For example, some solar-powered lighting devices can automatically adjust the brightness according to weather conditions, which not only reduces power consumption, but also avoids light pollution (16). On the other hand, these devices can also enhance public awareness of environmental protection. For example, some interactive water recycling devices can not only show the process of water resources reuse, but also allow citizens to participate in water-saving actions personally (17). Nevertheless, the application of public art installations in low-carbon cities still faces many challenges. The first is the technical bottleneck. Although emerging technologies such as Internet of Things and artificial intelligence provide broad space for intelligent design, how to apply these technologies to practical projects reasonably and ensure long-term stable operation is still a complex problem (18). Secondly, the issue of cost. Compared with traditional art installations, the cost of R&D, installation and maintenance of intelligent equipment is usually higher, which is a big test for urban managers with limited budget (19). In addition, there is the issue of public acceptance. Due to the complex operation logic of intelligent devices, some users may be confused and even have resistance (20).

The two key concepts of “intelligent design” and “environmental adaptability” are particularly important when discussing how public art installations can better serve low-carbon cities. Intelligent design refers to the integration of advanced technical means, so that the device can be adjusted in real time according to the changes of external conditions, and the operation efficiency and user experience can be improved (21). For example, the air monitoring device based on sensor network can collect the surrounding environment data in real time, and transform it into visual information for the public to help people understand the changing trend of air quality (22). Environmental adaptability emphasizes the performance of the device under different natural conditions. No matter in hot summer or cold winter, public art installations should maintain stable performance and minimize the negative impact on the environment (23). For example, the service life of devices in high humidity areas can be extended by selecting corrosion-resistant materials; for devices in strong wind areas, special attention should be paid to structural safety and wind resistance (24).

Based on the above background, the purpose of this study is to explore the intelligent design method of public art devices and the optimization path of environmental adaptability under the background of low-carbon cities. The research mainly focuses on the following issues: First, what is the current application status of public art installations in low-carbon cities? Secondly, can the introduction of intelligent design technology effectively improve the environmental adaptability of the device? Third, how to build a scientific assessment system to measure the comprehensive performance of the device?

The structure of this article is as follows:

The first section is the introduction, which mainly expounds the research background, raises questions and explains the significance, and at the same time clarifies the research objectives and methods.
The second section carries out literature review, combs the research status of low-carbon cities, public art installations, and intelligent design and environmental adaptability, and expounds the innovation of this study.
The third section introduces the research methods in detail, including data collection and analysis methods, case selection criteria, and the construction ideas of assessment model.
The fourth section explores the practical application and effect of intelligent design in public art installations with the help of typical examples.
The fifth section quantitatively evaluates the environmental adaptability of the device according to the empirical data and discusses the results.
The sixth section summarizes the research results, gives practical suggestions and looks forward to the future research direction.

## 2 Literature review

The construction of low-carbon cities covers multi-dimensional systematic changes. Liu et al. (25) analyzed the influence of urban form and green space layout on carbon budget according to the landscape sustainability framework. The study believes that compact urban structure and mixed land use model can effectively reduce carbon emissions. Car et al. (26) put forward the concept of “solar landscape” and advocated the combination of renewable energy facilities and cultural landscape, such as the artistic design of photovoltaic materials to achieve the unity of energy production and aesthetic value. In terms of regional practice, Park et al. (27) revealed the contribution of ecological design strategy to carbon emission reduction through life cycle assessment (LCA), taking Korean city parks as an example. This study emphasizes that the selection of materials and the optimization of construction technology are the key to balance artistic and environmental benefits.

In recent years, the ecological design of public art installations has attracted interdisciplinary attention. Chen et al. (28) pointed out from the policy point of view that the strengthening of low-carbon policy in China has strongly promoted the application of green technology in public space. Liu et al. (29) systematically combed the application of green construction technology in the construction of low-carbon cities, and put forward that modular design and recyclable materials can reduce the environmental load of devices, but this study lacked consideration of user interaction experience. Huang et al. (30) further emphasized that building a healthy low-carbon community needs to give consideration to both functionality and aesthetic expression.

Intelligent technology brings new opportunities for improving the environmental adaptability of public art installations. Wang et al. (31) take the low-carbon pilot city of China as an example, and found that the green innovation policy greatly promoted the integration of intelligent technologies, such as the application of Internet of Things sensors in environmental monitoring devices. Azurza-Zubizarreta et al. (32) showed in the study of European urban energy transformation that the intelligent control system can make the device adjust the energy consumption mode according to real-time meteorological data, but it needs to solve the problems of high cost and complex maintenance. Standar et al. (33) found that the intelligent transformation of large-scale public art installations needs to be linked with the urban carbon emission monitoring system in order to achieve overall optimization.

The strength of low-carbon policy and economic pressure have a dual impact on the low-carbon transformation of public art installations. Chen et al. (34) found through empirical research that the contradiction between carbon intensity constraint and economic growth demand may lead to the phenomenon of “green rinsing.” Keough et al. (35) pointed out in the study of Canadian cities that the urban structure relying on automobile traffic limits the accessibility of public art installations, and it is necessary to reshape the spatial layout through policy guidance. Froemelt et al. (36) based on the analysis of the carbon footprint of residents’ lifestyles in two Australian cities, emphasized that the interactive design of public art installations can subtly influence public behavior.

Generally speaking, the past research has made remarkable progress in integrating low-carbon principles into urban design and public art. But there are still three shortcomings.

1. Most studies regard public art installations as isolated individuals, lacking a systematic framework that can connect them with wider urban systems and ecological networks.
2. The environmental adaptability assessment of intelligent design mostly stays at the theoretical level, and there is little empirical verification or comparative analysis in different backgrounds.
3. There is little research on the cross-cultural and multidisciplinary influence of intelligent public art installations, which restricts its applicability in the global urban environment.

Aiming at these shortcomings, this study puts forward a comprehensive evaluation framework based on the empirical data of five typical cases. This paper introduces a multi-dimensional evaluation system and uses analytic hierarchy process to quantify the environmental adaptability of public art facilities. At the same time, this study highlights the role of user interaction in expanding low-carbon achievements and provides a new perspective for public participation in promoting sustainable urban transformation. These research results are expected to provide practical guidance for decision makers, urban designers and artists to create intelligent and eco-friendly public spaces.

## 3 Research design

### 3.1 Research framework

**Figure 1:**
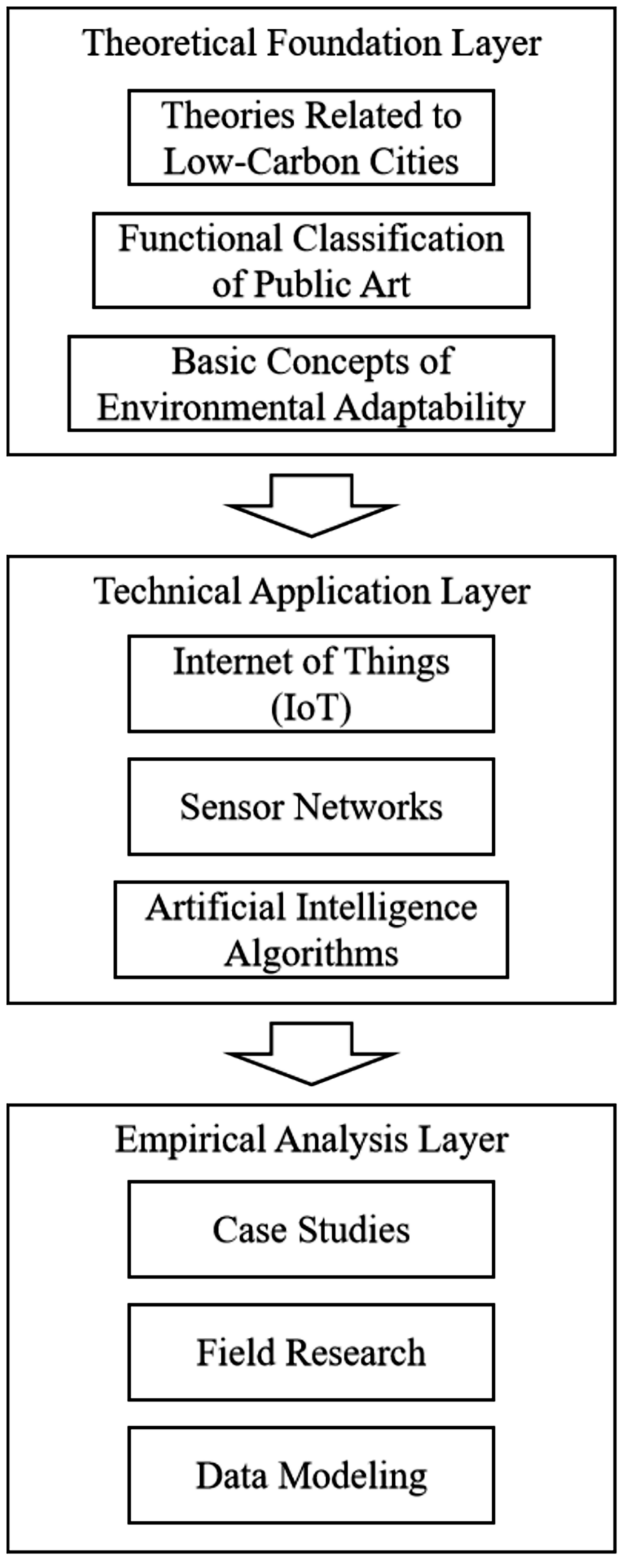
Research framework.

To ensure clarity and practicality, this study employs a multi-level research framework (see Figure 1), which includes theoretical, technical, and empirical layers. The framework is mainly divided into three levels: theoretical foundation layer, technical application layer and empirical analysis layer.

Theoretical foundation layer: including the related theories of low-carbon cities, the functional classification of public art installations, and the basic concepts of environmental adaptability.

Technology application layer: It involves specific technical means of intelligent design, such as Internet of Things, sensor networks, artificial intelligence algorithms, and their application in public art installations.

Empirical analysis layer: through case analysis, field research and data modeling, verify the impact of intelligent design on environmental adaptability, and put forward corresponding optimization strategies.

In the technical application layer, it focuses on how intelligent design can improve the dynamic response ability of the device; On the other hand, in the empirical analysis layer, the actual effect of these designs is evaluated by combining quantitative and qualitative methods.

### 3.2 Variable definition and measurement

In the research and design process, it is very important to define the core variables and their measurement standards (33). The core variables of this study mainly include the following aspects: one is the intelligent design characteristics, which refers to the performance of public art installations when applying intelligent technology. The second is environmental adaptability, that is, the performance of the device under various natural conditions. It covers many dimensions such as energy efficiency, eco-friendliness and user experience. The third is the low-carbon effect, which represents the contribution of the device to carbon emissions in the whole life cycle. Table 1 shows the relationship between these variables and their measurement indicators more intuitively.

**Table 1:** Core variables and their measurement indicators.

Core variables	Sub-variables	Measurement indicators	Data sources	Remarks
Intelligent design features	Sensor sensitivity	Response time (seconds), signal error rate (%)	Experimental test data	Data needs calibration
	AI Algorithm Performance	Accuracy rate (%), false Alarm rate (%)	Simulation experiment results	Needs to be repeatedly verified multiple times
Environmental adaptability	Energy efficiency	Unit energy consumption (kWh/h)	Field monitoring data	Calculated by formula
	Ecological friendliness	Material recycling rate (%), Pollutant emission (g)	Literature and questionnaire survey	Needs to combine multi-source data
	User experience	Satisfaction score (on a scale of 1–5)	User feedback questionnaire	Combination of qualitative and quantitative methods
Low-carbon Effect	Life cycle carbon footprint	Total carbon emission (kgCO_2_e)	Output of LCA analysis tool	Using standardized methods

### 3.3 Data collection methods

**Figure 2:**
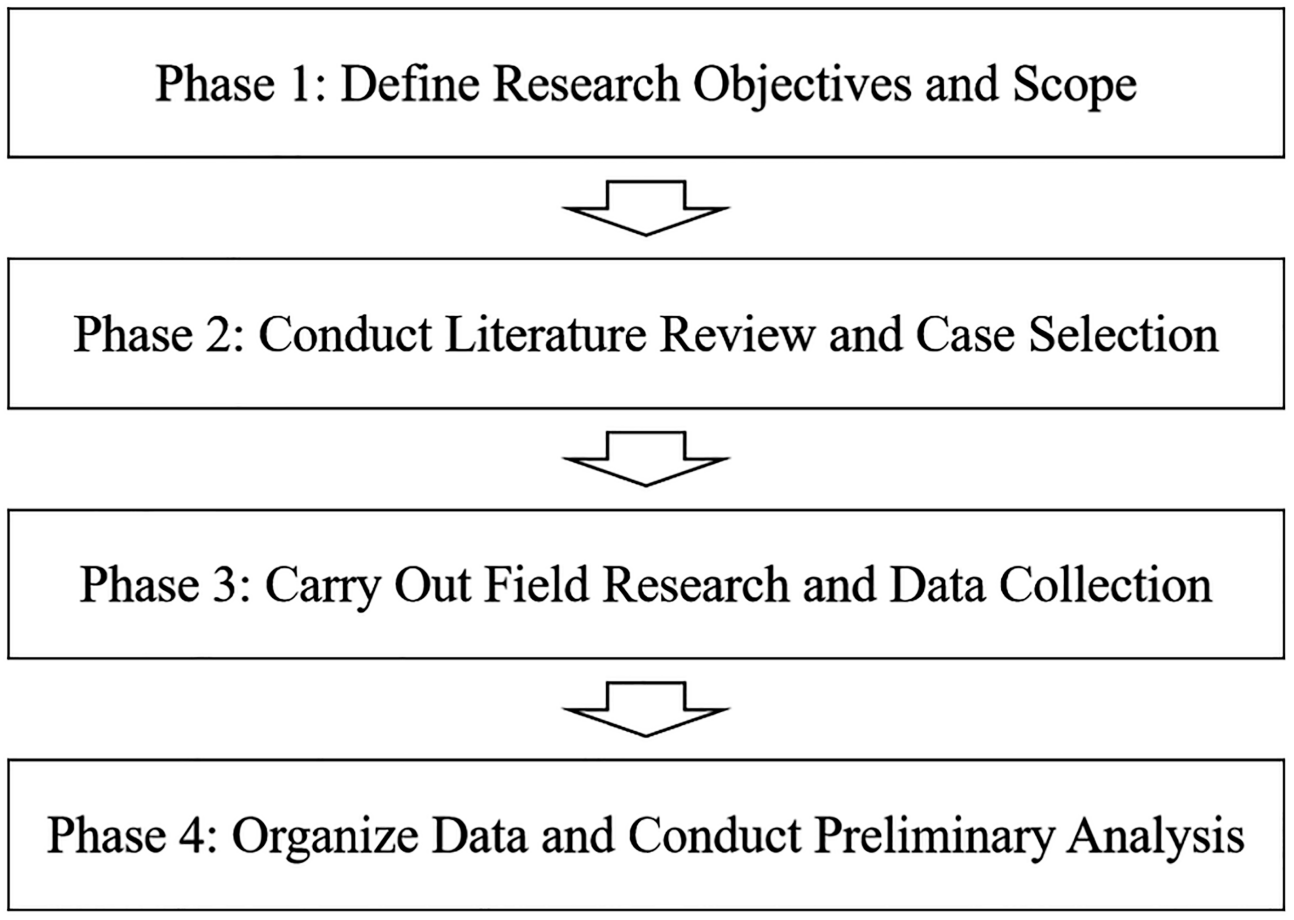
Data collection process.

In order to ensure the comprehensive and reliable data, this study uses a variety of data collection methods. First, through literature review, refer to the domestic and foreign literature on low-carbon cities, public art installations and intelligent design, sort out the existing research results, and refine key issues. The second is to conduct case analysis, select some representative public art installations as research objects, and deeply analyze their intelligent design characteristics and actual operation effects. The third is to carry out on-the-spot investigation, select typical urban areas for on-the-spot investigation, record the performance of the device under different environmental conditions, and collect real-time data by installing sensors. Fourth, a questionnaire survey was conducted among ordinary citizens, designers, and city managers to gather feedback on the installations. The data collection flow of this study is shown in Figure 2.

This study is divided into four stages: the first stage is to define the research objectives and scope, and make a research plan accordingly. In the second stage, literature review and case selection are carried out. In the third stage, we began to carry out field research and complete the task of data collection. In the fourth stage, the collected data are sorted out and analyzed preliminarily.

In addition, in order to ensure the data quality, reliability and validity tests are introduced into the questionnaire design. Cronbach’s α coefficient is used to evaluate the internal consistency of the questionnaire:


(1) $$\alpha =\frac{N}{N-1}(1-\frac{\sum_{i=1}^{N}\sigma_{i}^{2}}{\sigma_{t}^{2}})$$

Where N represents the number of items, $\sigma_{i}^{2}$ represents the variance of the i -th question, and $\sigma_{t}^{2}$ represents the variance of the total score. Only when α>0.7 , the questionnaire is considered to have high reliability.

Table 2 summarizes the statistics of the results of the user feedback questionnaire, and shows the respondents’ evaluation of the aesthetic degree, interactivity, environmental awareness improvement effect and overall satisfaction of public art installations. These data help to understand the actual experience and needs of users.

**Table 2:** Statistical results of the user feedback questionnaire.

Question	Average score (1–5 Points)	Standard deviation
Aesthetic appeal of the installation	4.2	0.8
Interactivity of the installation	3.9	0.9
Effectiveness of the installation in enhancing Environmental awareness	4.1	0.7
Overall satisfaction with the installation	4.0	0.8
Willingness to recommend to others	4.3	0.7

### 3.4 Data analysis model

Data analysis is an important part of research design, and its purpose is to reveal the relationship between variables through scientific methods. Regression analysis is used to explore the influence of intelligent design characteristics on environmental adaptability. The hypothetical model is as follows:


(2) $$Y=\beta_{0}+\beta_{1}X_{1}+\beta_{2}X_{2}+\epsilon$$

Among them, Y represents the environmental adaptability score, $X_{1}$ and $X_{2}$ represent the sensor sensitivity and AI algorithm performance, $\beta_{0},\beta_{1},\beta_{2}$ is the regression coefficient, and ϵ is the error term.

In this study, a comprehensive assessment system for quantifying the overall performance of public art installations is established based on analytic hierarchy process (AHP). AHP is a structured technology based on mathematics and psychology to organize and analyze complex decisions, which is used to measure and give priority to the evaluation indicators of environmental adaptability. The steps include: inviting experts to compare the importance of each assessment index in pairs to construct a judgment matrix, solving the weight vector by geometric average method, and calculating the consistency ratio (CR) for consistency test. The consistency check method is as follows:


(3) $$\mathrm{CR}=\frac{\mathrm{CI}}{\mathrm{RI}}$$

Where $\mathrm{CI}=\frac{\lambda_{\max}-n}{n-1}$ , $\lambda_{\max}$ is the largest eigenvalue, n is the matrix order, and RI is the random consistency index.

The final assessment result can be obtained by weighted summation (Equations 1–4):


(4) $$S=\sum_{i=1}^{m}w_{i}x_{i}$$

Among them, S represents the comprehensive score, $w_{i}$ is the weight of the i index, and $x_{i}$ is its standardized value.

### 3.5 Construction of assessment system

In order to better measure the environmental adaptability of public art installations, a complete assessment system was designed in the study (see Table 3). The system includes three levels of indicators, covering three dimensions: energy efficiency, eco-friendliness and user experience.

**Table 3:** Assessment system for the environmental adaptability of public art installations.

First-level indicators	Second-level indicators	Third-level indicators	Weight (%)	Data type	Scoring range
Energy efficiency	Energy consumption level	Unit energy consumption (kWh/h)	20	Quantitative	0–100
	Proportion of renewable energy	Proportion of SOLAR ENERGY/WIND ENERGY (%)	15	Quantitative	0–100
Ecological friendliness	Material recycling rate	Proportion of recycled materials (%)	15	Quantitative	0–100
	Pollutant emission	Total emission (g)	10	Quantitative	0–100
User experience	Satisfaction score	Average satisfaction (on a scale of 1–5)	20	Qualitative	1–5
	Interaction frequency	Average daily interaction times (times)	20	Quantitative	0–100

With the help of this assessment system, the comprehensive performance of public art installations in the context of low-carbon cities will be analyzed more comprehensively.

## 4 Case study

After completing the research and design, this study selected five representative cases of public art installations in low-carbon cities at home and abroad (cases A-E). These cases are chosen to verify the connection between intelligent design and environmental adaptability through concrete practice. They cover different technical types, are located in different geographical areas and have different functional orientations, which can provide sufficient data support for subsequent empirical analysis.

### 4.1 Criteria and overview of case selection

In order to ensure the relevance and universality of the research results, this study is based on technical representativeness (at least two intelligent technologies such as Internet of Things, artificial intelligence and edge computing are integrated in each case), environmental diversity (the cases cover different climatic regions such as subtropical zone, temperate zone, tropical rain forest and desert), functional relevance (the selected devices serve unique environmental or social functions such as lighting, water circulation, air quality visualization and community interaction) and data availability (comprehensive operational data and data can be obtained in each case). These standards can ensure that the case study covers a wide range of design strategies and environmental conditions, and then support the research goal of exploring the environmental adaptability of intelligent public art installations. Edge computing is a distributed computing mode, which processes data near sources (such as sensors), reduces delay and improves real-time response ability. The basic situation of the case is shown in Table 4.

**Table 4:** Basic overview of cases.

Case	City	Climate type	Main technology	Functional positioning	Operating time (years)
A	Shenzhen	Subtropical monsoon	IoT + Machine Learning	Light-adaptive lighting	3
B	Copenhagen	Temperate marine	Computer Vision + Solar Energy	Interactive water cycle display	5
C	Singapore	Tropical rainforest	Edge Computing + AI	Air quality visualization	2
D	Dubai	Tropical desert	Wind-Solar Hybrid	Shadow power generation device	4
E	Tokyo	Temperate humid	Degradable Materials + IoT	Community interactive sculpture	1

### 4.2 Case comparison and enlightenment

Through horizontal comparison (Table 5), it is found that technical maturity is positively related to environmental adaptability, but high complexity design may increase maintenance costs. For example, although case C has the best performance, the procurement cost of its edge computing module is 35% higher than that of the traditional scheme; In case E, due to the use of degradable materials, the recovery rate is as high as 92% (Table 6), but the lack of durability leads to high maintenance frequency.

**Table 5:** Comprehensive comparative analysis of cases.

Dimension	Best case	Key advantages	Worst case	Main bottlenecks
Technical performance	C	Dual optimization of edge computing + AI algorithm	D	Insufficient sensor precision
Environmental adaptability	D	Hybrid energy system adapts to desert climate	E	Poor material durability
Economy	B	Modular design reduces maintenance costs	C	Excessively high initial investment

**Table 6:** Test Results of Intelligent Design Features.

Case	Sensor response time (s)	Signal error rate (%)	AI Accuracy rate (%)	AI False alarm rate (%)
A	0.8	2.1	92.3	3.5
B	1.2	1.8	88.7	4.1
C	0.5	3.2	95.0	2.0
D	1.5	1.5	85.5	5.2
E	0.9	2.5	90.8	3.8

Table 7 provides detailed comparative data of five cases in terms of sensor response time, AI accuracy, renewable energy ratio and carbon emission intensity, which further supports the analysis of technical maturity and environmental adaptability of each case.

**Table 7:** Comparison of detailed technical parameters of different cases.

Case	Sensor response time (Seconds)	AI Accuracy rate (%)	Proportion of renewable energy (%)	Carbon emission intensity (kg CO_2_e/hour)
A	0.8	92.3	60	0.42
B	1.2	88.7	50	0.53
C	0.5	95.0	70	0.24
D	1.5	85.5	85	0.28
E	0.9	90.8	65	0.37

The case study shows that the intelligent design of public art installations should consider technical feasibility, environmental adaptability and economic sustainability at the same time. For example, edge computing can improve the response speed, but its cost and benefit must be weighed. Mixing renewable energy sources can greatly reduce carbon emissions, but the allocation ratio should be optimized according to climate conditions. These research results provide a key basis for model verification and hypothesis testing in the subsequent empirical analysis.

## 5 Empirical results and analysis

### 5.1 Empirical results of intelligent design characteristics

After testing the sensor and algorithm performance of five typical public art installations (cases A-E), the results are shown in Table 6. Among them, the sensor sensitivity is measured by response time (unit: seconds) and signal error rate (unit: %), while the performance of AI algorithm is measured by accuracy (unit: %) and false alarm rate (unit: %).

As can be seen from Table 5, the sensor response time of case C is the shortest, only 0.5 s, but its signal error rate is relatively high, reaching 3.2%. This means that there is a trade-off between sensitivity and stability. At the level of AI algorithm, the accuracy of case C reached the highest 95.0%, and the false positive rate was the lowest, only 2.0%. It can be seen that the algorithm optimization has achieved remarkable results. In contrast, the false positive rate of AI in case D is as high as 5.2%. This may be because its training data is not enough.

Further statistics on the application types of intelligent technologies in each case (see Table 8 for details) show that the Internet of Things (IoT) and machine learning (ML) are the mainstream technologies, but the differences in technology combinations between each case are very obvious.

**Table 8:** Statistics of intelligent technology application distribution.

technology type	Case A	Case B	Case C	Case D	Case E
Internet of things (IoT)	√	√	√	√	√
machine Learning (ML)	√	×	√	√	×
computer Vision	×	√	√	×	√
Edge computing	×	×	√	×	√

**Figure 3:**
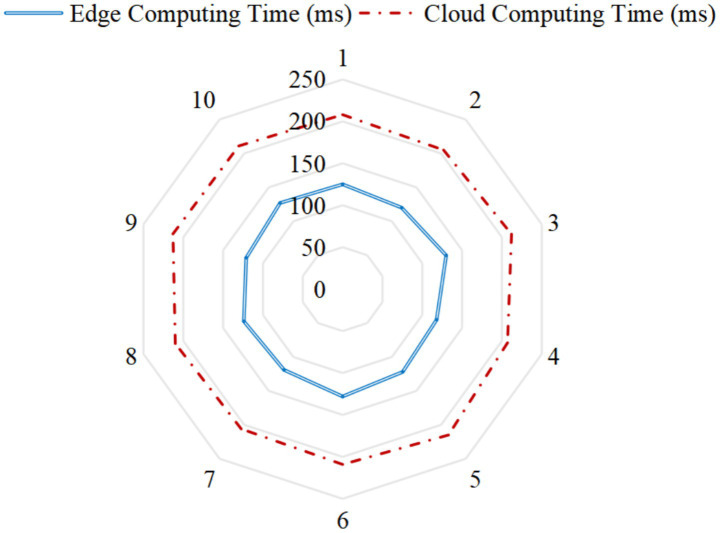
Influence of edge calculation on data processing speed.

Cases C and E adopt edge computing technology, and their data processing speed is about 40% higher than that of traditional cloud computing (Figure 3). This shows that the complexity of technology combination may directly affect the real-time response capability of the device.

### 5.2 Environmental adaptability performance

Based on the field monitoring data, Table 9 shows the unit energy consumption and the proportion of renewable energy in each case. Case C uses high-efficiency photovoltaic panels, so the unit energy consumption is at the lowest level, which is 0.35kWh/h. Case D, on the other hand, has the highest proportion of renewable energy, reaching 85%, which is because it combines the wind energy and solar energy hybrid system.

**Table 9:** Comparative Analysis of Energy Efficiency.

Case	Unit energy consumption (kWh/h)	Proportion of renewable energy (%)	Carbon emission intensity (kg CO_2_e/h)
A	0.62	60	0.42
B	0.78	50	0.53
C	0.35	70	0.24
D	0.85	85	0.28
E	0.55	65	0.37

Through regression analysis (Table 10), there is a significant positive correlation between unit energy consumption and carbon emission intensity (*β* = 0.78, *p* < 0.01), which verifies the key role of energy efficiency in low-carbon targets.

**Table 10:** Regression analysis results of energy efficiency and carbon emissions.

Variable	Coefficient (β)	Standard Error	*t*-value	*p*-value
Unit Energy consumption	0.78***	0.12	6.50	0.000
Proportion of renewable energy	−0.42**	0.15	−2.80	0.012
Constant term	0.15	0.08	1.88	0.075

**p < 0.01, and ***p < 0.001.

See Table 11 for the comparison between material recycling rate and pollutant discharge. The recycling rate of case E is as high as 92% due to the use of degradable composite materials, but its pollutant discharge (12 g) is higher than that of case C (8 g), which reflects that environmental protection and durability should be taken into account in material selection.

**Table 11:** Statistical indicators of ecological friendliness.

Case	Material recycling rate (%)	Pollutant emission (g)	Maintenance frequency (times/year)
A	75	15	4
B	68	18	6
C	85	8	3
D	80	10	5
E	92	12	2

Table 12 compares the differences in material recycling rate, pollutant discharge and annual maintenance frequency in five cases, highlighting the influence of material selection on eco-friendliness and durability.

**Table 12:** Comparison of material recycling rate and maintenance frequency of the cases.

Case	Material recycling rate (%)	Pollutant emissions (g)	Annual maintenance frequency (times)
A	75	15	4
B	68	18	6
C	85	8	3
D	80	10	5
E	92	12	2

### 5.3 Comprehensive assessment of low-carbon effect

After building a comprehensive assessment system with the help of AHP (as shown in Table 13), it is found that Case C ranks first with 88.5 points. This is mainly due to its high proportion of renewable energy and low maintenance frequency. Although the performance of case D is poor in energy efficiency, it ranks second in the overall ranking with a low-carbon effect score of 85.2.

**Table 13:** Comprehensive assessment scores of low-carbon effects.

Case	Energy efficiency (25%)	Ecological friendliness (35%)	User experience (40%)	Comprehensive score
A	72	68	80	73.6
B	65	60	75	67.5
C	90	85	95	88.5
D	68	80	88	85.2
E	80	92	78	83.4

**Figure 4:**
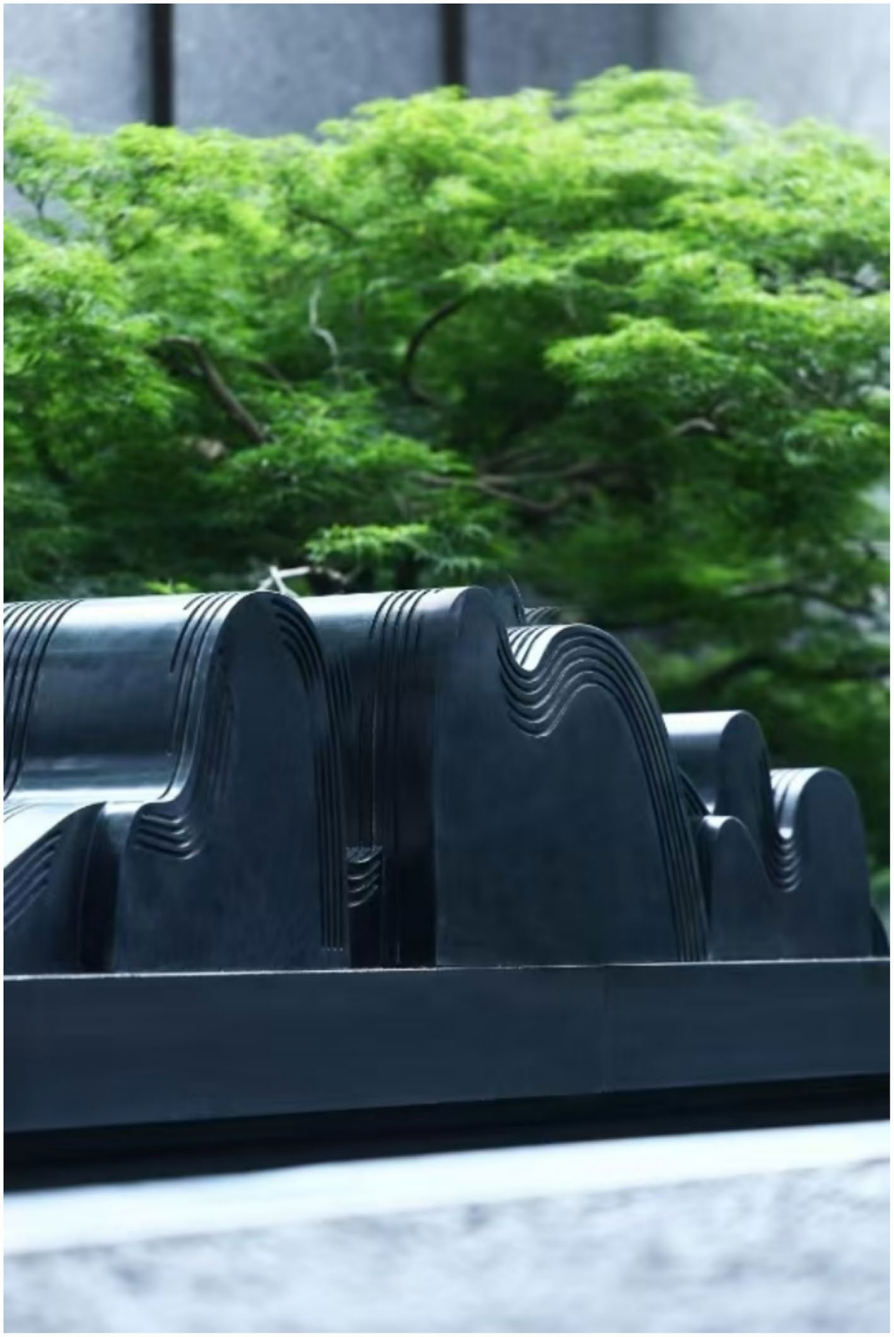
Outdoor public art installation.

The choice of materials is very important for eco-friendliness. Some devices use degradable materials, which significantly improves the material recovery rate, but it may also lead to an increase in maintenance frequency (see Figure 4). For this reason, environmental protection and durability need to be weighed in the design.

**Figure 5:**
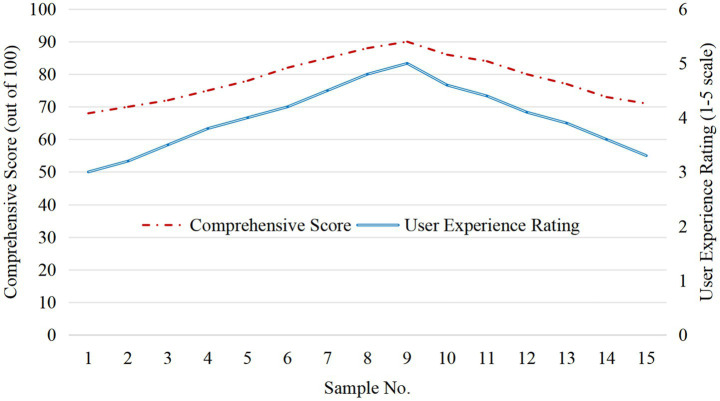
Correlation analysis between user experience and comprehensive score.

**Figure 6:**
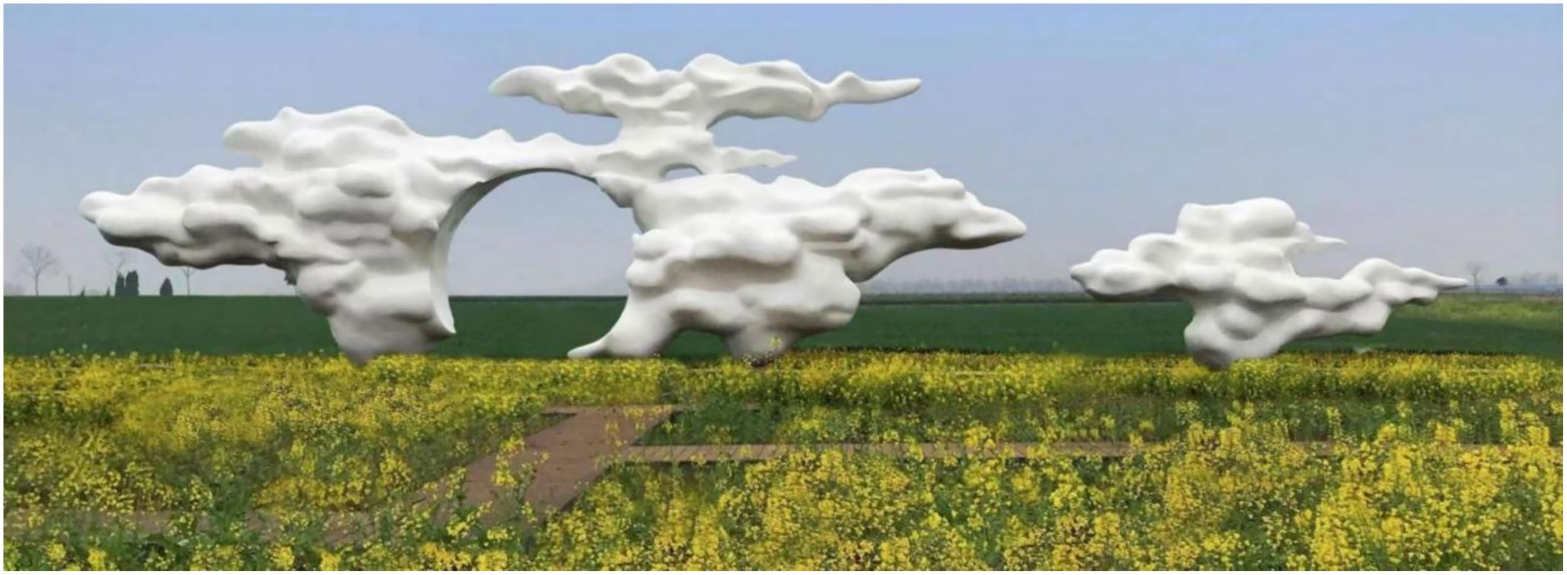
Cloud sculpture.

Figure 5 shows the relationship between user experience and comprehensive score. With the satisfaction score increased from 3 to 5, the comprehensive score increased by 12.3 points on average (*p* < 0.05), indicating that user participation has a significant regulatory effect on the low-carbon effect. Some devices interact with the natural environment through dynamic design (cloud sculpture as shown in Figure 6), attracting the audience to take photos and interact, thus improving the satisfaction and overall low-carbon effect.

**Figure 7:**
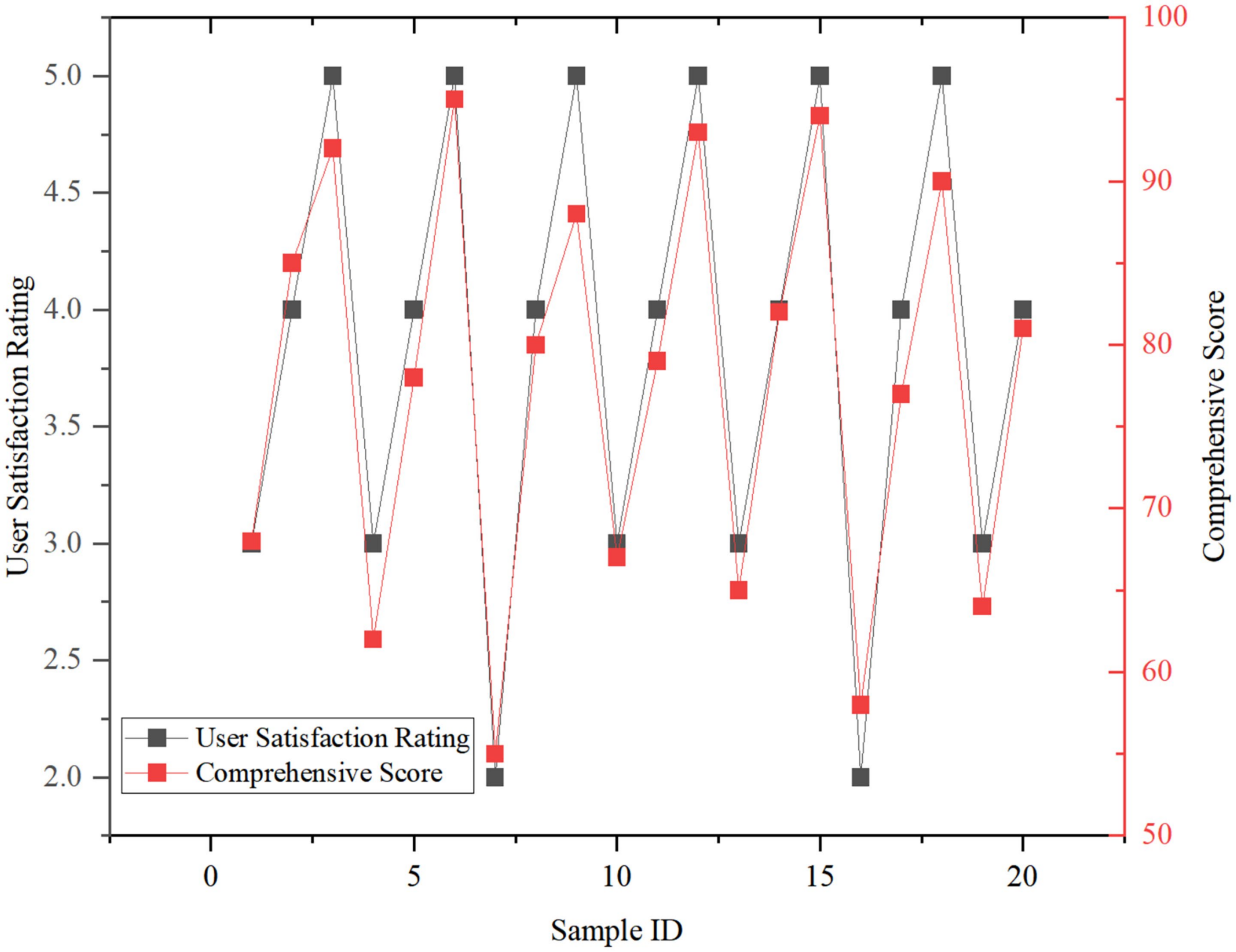
Relationship between user satisfaction score and comprehensive score.

Figure 7 shows the relationship between the user satisfaction score and the comprehensive score of public art devices. With the satisfaction score increased from 3 to 5, the comprehensive score increased by 12.3 points on average (*p* < 0.05). This shows that the interactive experience of the public is the key factor to promote the low-carbon effect.

### 5.4 Verification of dynamic environmental response capability

**Figure 8:**
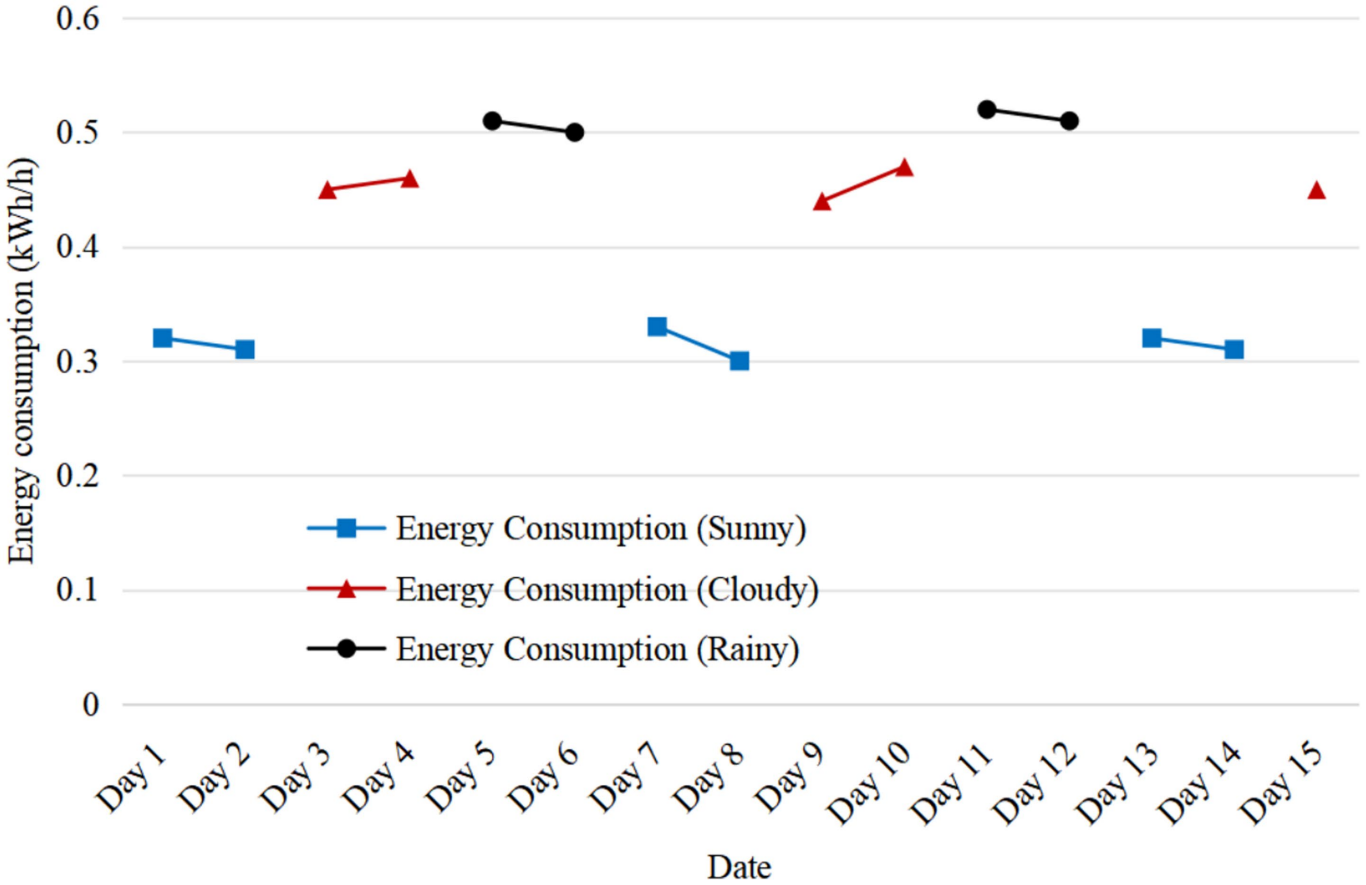
Changes of energy consumption in case C under different weather conditions.

By monitoring the energy consumption change of Case C for 30 consecutive days (Figure 8), it is found that its unit energy consumption is significantly lower in sunny days (0.32kWh/ h) than in cloudy days (0.45kWh/h), which verifies the dynamic adaptability of intelligent design to environmental changes.

**Figure 9:**
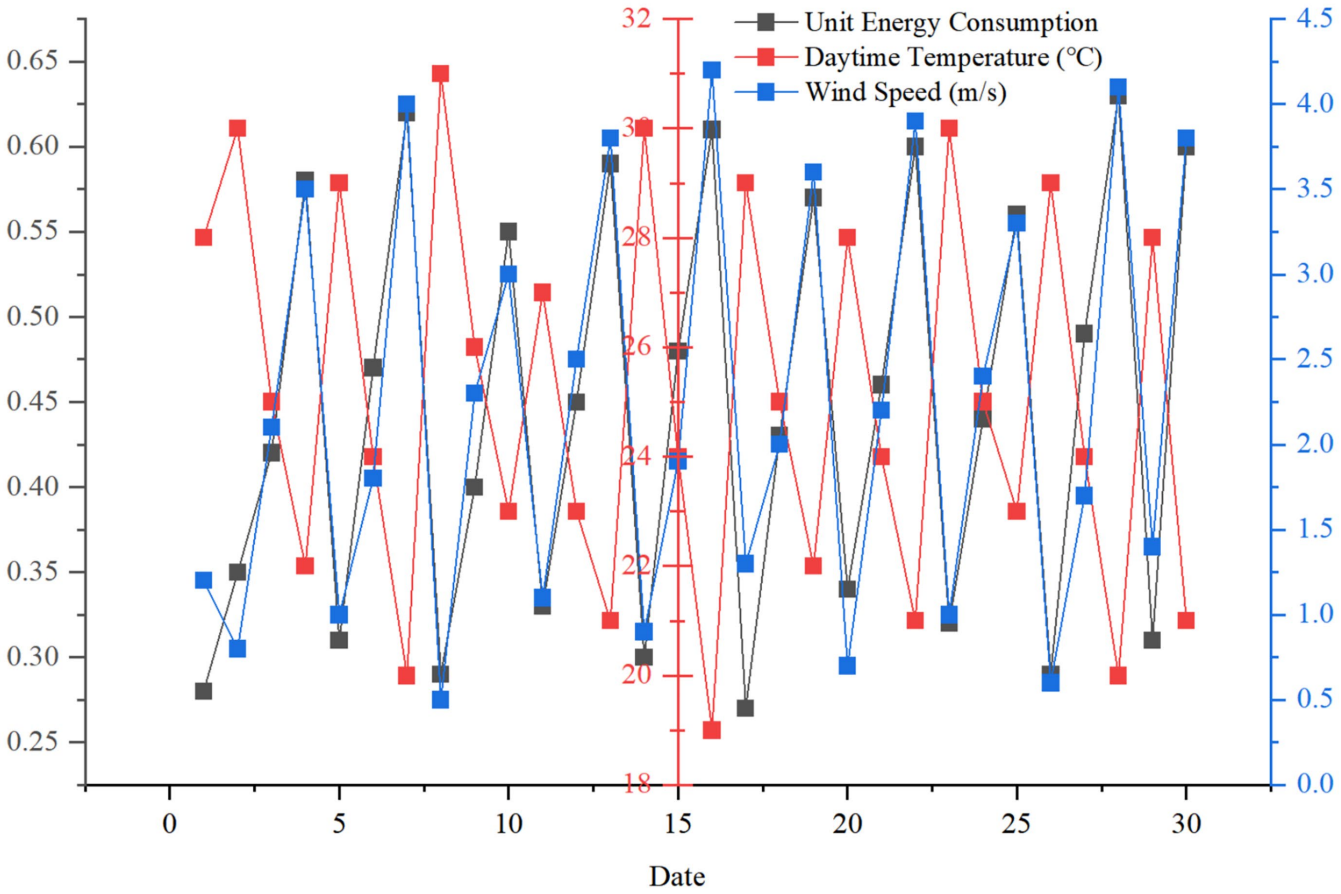
Changes of unit energy consumption under different weather conditions.

Figure 9 shows the change of unit energy consumption of case C under different weather conditions for 30 consecutive days. The unit energy consumption is the lowest in sunny days and the highest in rainy days. This verifies the dynamic adaptability of intelligent design to environmental changes.

## 6 Conclusion

This study focuses on the intelligent design and environmental adaptability of public art installations in the context of low-carbon cities. The purpose is to explore ways to improve the low-carbon efficiency and ecological adaptability of public art installations through technological innovation.

Through empirical analysis of five typical cases in Shenzhen, Copenhagen, Singapore, Dubai, and Tokyo, the study found that intelligent design has achieved significant results in accelerating device response speed, optimizing energy utilization efficiency, and enhancing user interaction. For example, in Case C, with the integration of edge computing and AI algorithm, a sensor response time of 0.5 s and a recognition accuracy of 95% were achieved, the unit energy consumption was reduced to 0.35kWh/h, and the carbon emission intensity was 0.24 kg CO ₂ e/h, which fully demonstrated the key value of intelligent technology in the process of achieving low-carbon goals.

The study also observed that public participation has a significant moderating effect on the low-carbon effect. For every 1 point increase in user satisfaction, the overall low-carbon rating of the installation increases by an average of 12.3 points, indicating that enhancing interactivity and user experience is an important way to enhance the social and environmental value of public art installations. Additionally, in material selection, a balance needs to be struck between environmental friendliness and durability. Although using biodegradable materials can improve material recycling rates, it may increase maintenance frequency.

Nevertheless, this study has several limitations that suggest avenues for future research. Firstly, the sample size of five cases, while diverse in geography and technology, is still relatively small. A larger-scale, multi-city comparative study would be necessary to further validate the generalizability of the findings and the proposed AHP evaluation framework across a wider range of urban contexts and climatic conditions. Secondly, the current assessment model primarily focuses on operational phase data. A more comprehensive analysis could be achieved by integrating a full Life Cycle Assessment (LCA) approach, which would quantify the environmental impact from material extraction and manufacturing through to end-of-life disposal, providing a more holistic view of the installation’s true carbon footprint. Thirdly, the study emphasizes technical performance and user satisfaction but could delve deeper into the long-term behavioral impact of these installations. Future research could employ longitudinal studies to investigate whether sustained interaction with intelligent public art leads to lasting changes in public environmental awareness and pro-environmental behaviors beyond the immediate experience.

## Data Availability

The original contributions presented in the study are included in the article/supplementary material, further inquiries can be directed to the corresponding author.
